# Frequency and patterns of second-line resistance conferring mutations among MDR-TB isolates resistant to a second-line drug from eSwatini, Somalia and Uganda (2014–2016)

**DOI:** 10.1186/s12890-019-0891-x

**Published:** 2019-07-10

**Authors:** David Patrick Kateete, Rogers Kamulegeya, Edgar Kigozi, Fred Ashaba Katabazi, Deus Lukoye, Sindani Ireneaus Sebit, Hergeye Abdi, Peter Arube, George William Kasule, Kenneth Musisi, Myalo Glen Dlamini, Derrick Khumalo, Moses L. Joloba

**Affiliations:** 1 Department of Immunology and Molecular Biology, School of Biomedical Sciences, Makerere University College of Health Sciences, Kampala, Uganda; 2 Department of Medical Microbiology, School of Biomedical Sciences, Makerere University College of Health Sciences, Kampala, Uganda; 3 National Tuberculosis/Leprosy Program, Ministry of Health Uganda, Kampala, Uganda; 4 WHO, Hargeisa, Somaliland Somalia; 5 Ministry of Health, Hargeisa, Somaliland Somalia; 6 World Vision International, Nairobi, Kenya; 7 National Tuberculosis Reference Laboratory, Kampala, Uganda; 8 National TB Reference Laboratory / eSwatini Health Laboratory Services, Ministry of Health, Hospital Hill Mbabane, Mbabane, eSwatini

**Keywords:** Tuberculosis, Fluoroquinolones, Aminoglycosides, *gyrA/gyrB*, *rrs*, *16S rRNA*, eSwatini, Somalia, Uganda

## Abstract

**Background:**

Pulmonary tuberculosis is a leading cause of morbidity and mortality in developing countries. Drug resistance, a huge problem in this contagious disease, is driven by point mutations in the *Mycobacterium tuberculosis* genome however, their frequencies vary geographically and this affects applicability of molecular diagnostics for rapid detection of resistance. Here, we report the frequency and patterns of mutations associated with resistance to second-line anti-TB drugs in multidrug-resistant (MDR) *M. tuberculosis* isolates from eSwatini, Somalia and Uganda that were resistant to a second-line anti-TB drug.

**Methods:**

The quinolone resistance determining region (QRDR) of *gyrA*/*gyrB* genes and the drug resistance associated fragment of *rrs* gene from 80 isolates were sequenced and investigated for presence of drug resistance mutations. Of the 80 isolates, 40 were MDR, of which 28 (70%) were resistant to a second-line anti-TB injectable drug, 18 (45%) were levofloxacin resistant while 12 (30%) were extensively drug resistant (XDR). The remaining 40 isolates were susceptible to anti-TB drugs. MIRU-VNTR analysis was performed for M/XDR isolates.

**Results:**

We successfully sub-cultured 38 of the 40 M/XDR isolates. The *gyrA* resistance mutations (Gly88Ala/Cys/Ala, Ala90Val, Ser91Pro, Asp94Gly/Asn) and *gyrB* resistance mutations (Asp500His, Asn538Asp) were detected in 72.2% (13/18) and 22.2% (4/18) of the MDR and levofloxacin resistant isolates, respectively. Overall, drug resistance mutations in *gyrA*/*gyrB* QRDRs occurred in 77.8% (14/18) of the MDR and levofloxacin resistant isolates. Furthermore, drug resistance mutations *a1401g* and *g1484 t* in *rrs* occurred in 64.3% (18/28) of the MDR isolates resistant to a second-line anti-TB injectable drug. Drug resistance mutations were not detected in drug susceptible isolates.

**Conclusions:**

The frequency of resistance mutations to second-line anti-TB drugs in MDR-TB isolates resistant to second line anti-TB drugs from eSwatini, Somalia and Uganda is high, implying that rapid molecular tests are useful in detecting second-line anti-TB drug resistance in those countries. Relatedly, the frequency of fluoroquinolone resistance mutations in *gyrB*/QRDR is high relative to global estimates, and they occurred independently of *gyrA*/QRDR mutations implying that their absence in panels of molecular tests for detecting fluoroquinolone resistance may yield false negative results in our setting.

**Electronic supplementary material:**

The online version of this article (10.1186/s12890-019-0891-x) contains supplementary material, which is available to authorized users.

## Background

Pulmonary tuberculosis (TB) remains a persistent global public health problem with a huge toll on populations in developing countries [1, 2]. The drug-resistant forms of the disease especially multidrug resistant TB (MDR-TB) and extensively drug-resistant TB (XDR-TB) pose significant challenges for control of pulmonary TB and management of TB patients [3]. MDR-TB is caused by *Mycobacterium tuberculosis* strains that are resistant to the two powerful first-line anti-TB drugs, rifampicin and isoniazid. Extensively drug-resistant TB is “a rare type of MDR-TB that is resistant to a fluoroquinolone (FQ) and any of the three second-line injectable drugs: amikacin (AMK), kanamycin (KAN), and capreomycin (CAP)” [4]. MDR-TB and XDR-TB are very difficult to treat as the drug regimens are lengthy, toxic, and expensive [5, 6]. TB control programs in many countries depend on early diagnosis and prompt identification of drug resistance [3, 7] and this is critical for reduction of resistance and preventing its spread in communities. Usually, the diagnosis of drug-resistant TB is achieved through sputum culturing accompanied by drug susceptibility testing (DST) of the bacilli, either in liquid or on solid culture media however, these conventional procedures are laborious, lengthy, expensive [7], and often not available in developing countries. As such, molecular susceptibility tests that can be performed within 48 hours are currently being deployed in African countries for rapid detection of resistance to anti-TB drugs, and the World Health Organization (WHO) has endorsed them for rapid detection of resistance to first-line and second-line anti-TB drugs [3]. Currently, the MTBDR*sl* test (Hain Lifescience, Nehren Germany) is the only available commercial test for rapid detection of resistance to second-line anti-TB drugs [8]; it targets resistance to FQs and second-line anti-TB injectable drugs AMK, KAN and CAP.

Spontaneous chromosomal point mutations are the main mechanism underlying drug resistance in TB [9] and a limited number of mutations accounts for majority of the phenotypic resistance to first- and second-line anti-TB drugs. Resistance to FQs, the most effective second-line anti-TB drugs used to treat MDR-TB, is associated with mutations in a short discrete region of the *gyrA* gene and less frequently *gyrB,* commonly referred to as the quinolone-resistance determining region (QRDR) [10]. Globally, up to 80% of FQ resistant *M. tuberculosis* is attributed to mutations in *gyrA*/QRDR mainly in codons 88, 90, 91 and 94 [10]. Furthermore, AMK, KAN and CAP resistance has been associated with mutations in the *16S rRNA* gene (*rrs*), between nucleotide positions 1400 and 1500 particularly mutations in positions 1401, 1402 and 1484; up to 87% of KAN and AMK resistance worldwide is attributed to these mutations (especially a1401g) [11, 12]. Additionally, KAN and CAP resistance can also be mediated by mutations in the *eis* and *tlyA* genes, respectively.It is now well-documented that the frequencies of drug resistance conferring mutations described above vary between *M. tuberculosis* geno-groups and geographically [13, 14], which pose a significant challenge in the development of sequence-based diagnostic tools and their applicability without a thorough evaluation of their performance with respect to setting. Case in point is the MTBDR*sl* test which, although it is generally useful in detecting resistance to second-line anti-TB drugs, it has varying sensitivity especially in detecting FQ resistance [15]. This creates need for characterizing *M. tuberculosis* isolates from different geographical regions to investigate frequencies of drug resistance conferring mutations. Here we describe these mutations in FQ (levofloxacin LXF, ofloxacin OFX, moxifloxacin MXF) resistant and AMK/KAN/CAP resistant *M. tuberculosis* isolates from eSwatini (formally Swaziland), Somalia and Uganda, all TB endemic countries with high prevalence of HIV/TB co-infection [16, 21].

## Methods

### Study setting and samples

This was a cross-sectional study on stored isolates at Makerere University College of Health Sciences in Kampala, Uganda. Samples (sputum or isolates) were obtained from ongoing national surveillances for MDR-TB and XDR-TB in the three countries of eSwatini, Somalia and Uganda. The isolates were cultured from patients with confirmed MDR-TB or presumptive XDR-TB in which phenotypic or genotypic resistance to at least a second-line anti-TB drug was confirmed in the period between September 2014 and July 2016, Additional file 1: Table S1. A total of 40 MDR-TB and/or presumptive XDR-TB isolates met this criteria: 15 from eSwatini, 13 from Uganda and 12 from Somalia. This sample size reflects the WHO reports for proportions of MDR/RR-TB cases that are tested for resistance to second-line drugs in those countries [22], see references [23, 25] for details on country specific profiles for TB [23–25]. Furthermore, 40 isolates that were susceptible to first- and second-line anti-TB drugs were also included bringing the total number of isolates investigated to 80 (all non-repetitive isolates, one per patient). Samples/isolates from eSwatini and Somalia were shipped to Uganda and sub-cultured at the Supra-National Tuberculosis Reference Laboratory (SRL) in Kampala as described previously [16, 18, 21].

### Drug susceptibility testing

Drug susceptibility testing (DST) was performed at the SRL in Kampala and repeated for second-line drug resistance at the Mycobacteriology (BSL-3) Laboratory, Makerere University College of Health Sciences. The isolates to be tested for resistance were sub-cultured for up to 14 days on Middlebrook 7H10 medium. Then, cultures were diluted to a McFarland standard of 0.5 by resuspending few healthy colonies in Middlebrook 7H9 medium with glycerol. We used the agar proportion method and antibiotic critical concentrations (CCs) recommended by the WHO for Middlebrook 7H10 medium i.e. LXF 1 μg/ml, MXF 0.5 μg/ml, AMK 2 μg/ml, CAP 4 μg/ml and KAN 4 μg/ml [26]. Middlebrook 7H10 agar quadrant plates were prepared by following standard microbiological procedures, and configured such that quadrant 1 was the drug free control while quadrants 2, 3 and 4 contained antibiotics at the respective CCs indicated above. Plates were inoculated by following standard microbiological procedures i.e. with 100 μl of 10^−2^ and 10^−4^ dilutions of inoculum adjusted to the turbidity equivalent to a McFarland standard of 0.5, and the inoculum spread uniformly on the plate by tilting it (gently). The plates were sealed in plastic bags and incubated at 37 °C for 21 days while being examined weekly. After 21-28 days of incubation, colonies on the plates were counted with the aid of a microscope. Antibiotic susceptibility was determined by comparing growth on drug containing quadrants to growth on drug free quadrant. An isolate was classified as resistant when colonies on the drug containing quadrant were more than 1% compared to the colonies on the drug free quadrant (control). Drug susceptible *M. tuberculosis* H37Rv strain and known M/XDR *M. tuberculosis* isolates were used for quality control.

### Genotyping

Chromosomal DNA used as template in PCRs was extracted by following the CTAB/chloroform extraction method [27] with minor modifications as described previously [14]. Briefly, freshly cultured *Mycobacterium* cells were harvested and re-suspended in absolute ethanol (Sigma scientific, USA); the suspension was centrifuged at 16,000 g and the cell pellet re-suspended in 0.25X Tris-EDTA (TE) buffer, which was used as template in PCRs. Prior to genotyping, isolates were confirmed to be *M. tuberculosis* by following an in-house PCR protocol described previously [28], in which they all tested positive for *M. tuberculosis* complex. To determine the genotypes, 15 loci Mycobacterial Interspersed Repetitive Units-Variable Number of Tandem Repeats (MIRU-VNTR) genotyping was performed as described previously [29].

### PCRs and DNA sequencing

The primers we used, and procedure we followed to amplify the targeted portions of genes associated with resistance to second-line anti-TB drugs i.e. *gyrA*/*gyrB* QRDR and *rrs* were previously reported [7] (summarized in Additional file 1: Table S1, sheet 2). Primers were synthesized by Eurofins Genomics, Germany. PCRs were performed according to the protocol for the HotStar PCR kit (QIAGEN, Germany), in 60 μL total volume as described previously [14]. Also, post-amplification analysis of the PCR products was as previously described [14]. Fifty microliters each of the PCR products was purified using the QIAmp DNA purification mini kit (QIAGEN) and sequenced by using the Sanger method at ACGT Inc. (Wheeling IL, USA).

### Sequence analysis and interpretation of mutations

The amplicon-sequences were analysed first, by BLAST-searching at https://blast.ncbi.nlm.nih.gov/Blast.cgi to confirm they match the expected sequences in the *M. tuberculosis* genome*.* Furthermore, the *gyrA*/*gyrB* amplicon sequences were translated into amino acid sequences using MEGA6.06 software [30] or ExPASy online server http://web.expasy.org/translate/. Additionally, amplicon sequences were aligned to the *M. tuberculosis* H37Rv RefSeq sequences (NCBI reference sequence NC_000962) for *gyrA, gyrB*, & *rrs* promoter, using MEGA6.06 or BioEdit v7.2.5.0. To identify and interpret the mutations and determine whether they were conferring resistance to second-line anti-TB drugs, we used the ReSeqTB database http://www.reseqtb.org/Account/Login?ReturnUrl=%2F and current literature [7, 8, 31]. As well, for *gyrB*, we used the “500-538” codon numbering system (1998) that most studies on FQ-resistance mutations have used, see Malik et al and Maruri et al, references [32, 33], respectively. The data was curated and presented as tables or percentages depending on frequencies of mutations that occurred in the examined sequences. *M. tuberculosis* strain H37Rv is susceptible to anti-TB drugs and its *gyrA, gyrB,* and *rrs* sequences were regarded the “wild-type” during the analyses.

### Quality control

Known FQ and KAN resistant MDR-TB isolates with well-characterized resistance conferring mutations were included as positive controls. Sequences were validated through nucleotide BLAST and translated BLAST searches to confirm that they match the expected genes in *M. tuberculosis.*

## Results

### Second-line anti-TB drug resistance

We successfully sub-cultured all the 40 drug susceptible isolates and 38 of the 40 MDR / presumptive XDR-TB isolates that were resistant to a second-line anti-TB drug. All the isolates were investigated for mutations associated with resistance to second-line anti-TB drugs i.e. FQs (LXF, OFX, MXF), aminoglycosides (KAN, AMK) and CAP (a cyclic peptide). However for FQ resistance, only LXF and MXF resistance were considered in the analysis given the WHO’s recommendation against testing for OFX resistance [26]. Generally KAN resistance was high among MDR isolates (60.5%, 23/38) but comparable with respect to country of origin, Additional file 1: Table S1. AMK and CAP resistance was also high at 58% (22/38) and 50% (19/38), respectively; as well, LXF/MXF resistance was high (47.4%, 18/38). Overall, 73.7% (28/38) of the isolates were resistant to a second-line anti-TB injectable drug, of which 12 (31.6%) were confirmed to be XDR as they were resistant to either LXF or MXF.

### *M. tuberculosis* genotypes

Thirty four of the 38 M/XDR isolates were successfully genotyped by MIRU-VNTR analysis. The 13 isolates from Uganda were *M. tuberculosis* sub-lineages Uganda II (four isolates), Beijing (two isolates), and one isolate each for genotypes Uganda I, Delhi/CAS, NEW 1, LAM, West Africa 1 and S; one isolate from Uganda could not be genotyped. The 10 isolates from Somalia were *M. tuberculosis* Uganda I, West Africa II and Beijing (two isolates each) and one isolate each for genotypes Uganda II, West Africa I, and S; also, one isolate from Somalia could not be genotyped. Likewise, genotypes for the 15 isolates from eSwatini were diverse with Beijing (four isolates) and LAM (two isolates) being the most prevalent, and one isolate each for TUR, S, EAI, West Africa 1, and NEW 1 –two isolates from eSwatini could not be genotyped, Additional file 1: Table S1. Overall, the most predominant genotypes in this study were *M. tuberculosis* Uganda (23.7%, 9/38) and Beijing (21%, 8/38), Additional file 1: Table S1. Genotypes for the XDR isolates were Beijing (three), NEW-1, Uganda II, S, West Africa 2 (two isolates each) and Delhi/CAS (one isolate). Genotypes for the 40 drug-susceptible isolates were described elsewhere [34, 35].

### Drug resistance conferring mutations

The most frequent drug resistance mutation in the MDR and LXF/MXF resistant isolates was Asp94Gly (33.3%) and it was not detected in drug susceptible isolates, Table 1. Overall, drug resistance mutations in *gyrA*/QRDR were detected in a total of 13 (72.2%) MDR and LXF/MXF resistant isolates i.e. Asp94Gly (six isolates), Ala90Val (three isolates) as well as Gly88Cys, Gly88Ala, Ser91Pro and Asp94Asn, each occurring in one isolate, Table 1; Additional file 1: Table S1. For *gyrB*/QRDR, drug resistance mutations were detected in four (22.2%) MDR and LXF/MXF resistant isolates i.e. Asp500His (3 isolates), Asn538Asp (1 isolate), Table 1 and Additional file 1: Table S1. Three of the four isolates (TC41789, TC84410 & TC84702) with resistance mutations in *gyrB* also possessed drug resistance mutations in *gyrA*/QRDR (Asp94Val / Ala90Val), Additional file 1: Table S1. Interestingly, isolate MG87552 from Somalia possessed only the mutation Asp500His and it lacked resistance mutations in *gyrA*/QRDR (Additional file 1: Table S1), implying that FQ resistance in this isolate was attributed to Asp500His. Additionally, all but one isolate with *gyrB* resistance mutations were from Uganda and all were *M. tuberculosis* Uganda genotype while the sole isolate from Somalia with a *gyrB* resistance mutation was *M. tuberculosis* West African 1. Overall, FQ resistance mutations either in *gyrA*/QRDR or *gyrB*/QRDR occurred in a total of 14 (77.8%) MDR and LXF/MXF resistant isolates, Additional file 1: Table S1. Interestingly, 5 (27.8%) MDR and LFX/MXF resistant isolates lacked known drug resistance mutations in *gyrA*/*gyrB* QRDRs. Almost all the MDR and LXF/MXF resistant isolates had mutations outside the *gyrA*/QRDR but they also occurred in LXF/MXF susceptible isolates though at lower frequencies, Table 1.

**Table 1:** Frequency and patterns of mutations associated with phenotypic resistance to second-line anti-TB drugs among MDR- and XDR-TB isolates resistant to a second-line TB drug from eSwatini, Somalia and Uganda (2014–2016)

Drug	Locus	Mutation	Frequency (No. of isolates):-							
			MDR & LXF/MXF resistant (*n* = 18)				LXF/MXF susceptible / Pan-susceptible (*n* = 40)			
			With mutation	Relative Frequency (%)	Without mutation	Relative Frequency (%)	With mutation	Relative Frequency (%)	Without mutation	Relative Frequency (%)
FLUOROQUINOLONES (LXF, MXF)	*gyrA*	Thr2Ala	05	27.8	13	72.2	0	0	40	100
		Thr2Arg	05	27.8	13	72.2	0	0	40	100
		Thr2Ala/Arg	10	55.6	08	44.4	0	0	40	100
		Asp3Glu	04	22.2	14	77.8	02	5	38	95
		Asp3Arg	03	16.7	15	83.3	01	2.5	39	98
		Asp3Val	04	22.2	14	77.8	0	0	40	100
		Asp3Glu/Asp3Ar/Asp3Val	11	61.1	07	40	0	0	40	100
		Asp3Arg	03	16.7	15	83.3	0	0	40	100
		Asp3Val	01	5.6	17	94.4	0	0	40	100
		**Gly88Cys**	01	5.6	17	94.4	0	0	40	100
		**Gly88Ala**	01	5.6	17	94.4	0	0	40	100
		**Gly88Ala/Cys**	02	11.1	16	89	0	0	40	100
		**Ala90Val**	**03**	**16.7**	**15**	**83.3**	**0**	**0**	**40**	**100**
		**Ser91Pro**	**01**	**5.6**	**17**	**94.4**	**0**	**0**	**40**	**100**
		**Asp94Gly**	**06**	**33.3**	**12**	**66.7**	**0**	**0**	**40**	**100**
		**Asp94Asn**	**01**	**5.6**	**17**	**94.4**	**0**	**0**	**40**	**100**
		**Asp94Asn/Gly**	**07**	**39**	**11**	**61.1**	**0**	**0**	**40**	**100**
		**Gly88Ala/Gly88Cys/Gly88Ala/Ala90Val/Ser91Pro/Asp94Gly/Asp94Asn/Asp94Gly** ^a^	**13**	**72.2**	**05**	**27.8**	**0**	**0**	**40**	**100**
		Arg128Ser	01	5.6	17	94.4	01	2.5	39	98
		Tyr129Asn	04	22.2	14	77.8	01	2.5	39	98
		Ala132Glu	02	11.1	16	89	0	0	40	100
	*gyrB*	Gln608His	01	5.6	17	94.4	0	0	40	100
		Arg609Gly	**02**	**11.1**	**16**	**89**	0	0	40	100
		Tyr610Thr	01	5.6	17	94.4	0	0	40	100
		Arg485His	01	5.6	17	94.4	0	0	40	100
		Lys611Gln	**03**	**16.7**	**15**	**83.3**	01	2.5	39	98
		**Asp500His**	**03**	**16.7**	**15**	**83.3**	0	0	40	100
		**Asn538Asp**	**01**	**5.6**	17	94.4	0	0	40	100
		**Asp500His/ Asn538Asp**	**04**	**22.2**	14	77.8	0	0	40	100
KAN CAP AMK	*rss*	MDR & KAN/CAP/AMK resistant (*n* = 28)					KAN/CAP/AMK susceptible / Pan-susceptible (*n* = 40)			
		**a1401g**	**17**	**60.7**	**11**	**39.3**	**0**	**0**	**40**	**100**
		**g1484 t**	**01**	**3.6**	**27**	**96.4**	**0**	**0**	**40**	**100**
		**a1401g/g1484 t** ^a^	**18**	**64.3**	**10**	**35.7**	**0**	**0**	**40**	**100**
		g1158 t	01	3.6	27	96.4	0	0	40	100

High confidence mutations are presented in boldface font

^a^Number of isolates with any of these high confidence mutations

By country, isolates with FQ resistance conferring mutations were distributed as follows: Uganda –six isolates with mutations *gyrA*/Ala90Val + *gyrB*/Asp500His (two isolates), *gyrA*/Asp94Gly + *gyrB*/Asn538Asp (one isolate), *gyrA*/Asp94Gly (two isolates) and *gyrA*/Ser91Pro (one isolate). Genotypes for these isolates were *M. tuberculosis* Uganda II (two isolates), Uganda I, West African 1, NEW-1 and Beijing (one isolate each). eSwatini –four isolates with mutations Asp94Gly, Asp94Asn, Asp94Gly, and Gly88Cys and their genotypes were Beijing (two isolates) and S (one isolate). Somalia –four isolates with *gyrA* mutations Gly88Ala, Asp94Gly, Ala90Val and *gyrB* mutation Asp500His; their genotypes were West African 2 (two isolates), S (one isolate) and Uganda I (the isolate with *gyrB*/Asp500His).

Furthermore, almost all the 28 MDR-TB isolates resistant to a second-line anti-TB injectable drug (KAN, CAP, AMK) had mutations in the *rrs* gene (96.4%, 27/28) with *a1401g* being the most frequent (60.7%, 17/28) while *g1484 t* occurred in only one isolate, Table 1. Altogether, the drug resistance mutations *a1401g* and *g1484 t* combined occurred in 18 (64.3%) MDR and KAN/CAP/AMK resistant isolates while 10 (35.7%) isolates lacked known drug resistance conferring mutations in *rss*. Similar to *gyrA*, the *rrs* mutations were more frequent in the Beijing genotype and by country, they were proportionally more frequent in isolates from eSwatini and Uganda. None of the 40 drug susceptible isolates harboured resistance conferring mutations to second-line anti-TB drugs. Of note, several lineage-specific polymorphisms and mutations that are not known to confer resistance to second-line drugs were detected but these are not discussed (Additional file 1: see Table S1).

## Discussion

In this study, the combined frequency of known drug resistance conferring mutations in MDR-TB isolates that were resistant to a second-line anti-TB drug from eSwatini, Somalia and Uganda was moderately high i.e. 64.3% for injectable drugs and 77.8% for FQs, and comparable to reported frequencies for these mutations worldwide [7, 31]. However, our rates are slightly lower compared to some reports [7, 8, 31, 36] but such discrepancies are commonly reported even in studies from the same region or country [37, 38]. Several investigators worldwide also reported low individual frequencies for mutations especially *gyrA*/Asp94Asn and *gyrA*/Asp94Gly in FQ resistant *M. tuberculosis* isolates e.g. 25% in Shanghai China [39] and 10% (2/20) in a recent study in Uganda [40]. In one systematic review of the gyrase mutations associated with FQ resistance in *M. tuberculosis*, only 780 (64%) of the 1220 FQ resistant isolates had mutations, of which those affecting the drug resistance *gyrA* codons 90, 91 or 94 were present in only 54% (654/1220) of the resistant isolates [33]. In India, only 17% (25/146) of the MDR isolates had mutations in *gyrA*/QRDR with the commonest being Asp94Gly (48.1%, 13/27) [41]. Generally, the observed low frequencies for individual resistance conferring mutations relative to global estimates could be attributed to mutations or resistance mechanisms in other key genes we did not investigate e.g. *eis* for second-line anti-TB injectable drugs [31].

In this study, the overall frequency (77.8%) of drug resistance conferring mutations in *gyrA* and *gyrB* of FQ resistant MDR-TB isolates was high and almost similar to global estimates for frequencies of these mutations in FQ resistant *M. tuberculosis* isolates i.e. 80%-92% [42, 43]. Interestingly, the frequency (22.2%) of resistance mutations Asp500His and Asn538Asp in *gyrB*/QRDR in this study was higher than global estimates for these mutations i.e. ≤2% [42] or ≤5% [43]. Generally, *gyrB*/QRDR resistance mutations especially Asn538Asp, Asp500His and Arg485His were confirmed to confer cross-resistance to FQs in *M. tuberculosis* [32] but they are considered to be rare and occur at much lower frequencies (i.e. 1% - 2% in FQ resistant *M. tuberculosis* isolates [42]). Whether *gyrB* resistance mutations are more frequent in African populations as suggested by this study requires further investigation.

Usually, drug resistance mutations in *gyrB*/QRDR occur in association with *gyrA*/QRDR resistance mutations and they mostly affect codons 500 and 538 [42, 43]. The association of *gyrB* resistance mutations with *gyrA* mutations has made it difficult to evaluate the contribution of *gyrB* mutations to FQ resistance [42]. Interestingly, in this study the *gyrB* mutation Asp500His occurred in a FQ resistant isolate independently of resistance mutations in *gyrA*/QRDR, implying that *gyrB* mutations in our setting individually confer resistance to FQs, as experimentally validated by Malik et al [32]. Relatedly, in a systematic review of the frequencies and geographic distribution of *gyrA* and *gyrB* mutations associated with FQ resistance in clinical *M. tuberculosis* isolates [42], it was reported that *gyrB* mutations indeed occurred independently of *gyrA* mutations in certain settings perhaps explaining phenotypic resistance in isolates lacking mutations in *gyrA*/QRDR [42].

Furthermore, we also detected mutations in *gyrA*/*gyrB* genes that are not drug resistance conferring e.g. Thr2Ala/Arg, Asp3Val, Thr4Gly, Ala132Asp, Ala132Glu and Arg133Gln, and they were not included in systematic surveys of confidence-graded mutations associated with phenotypic resistance to FQs [31]. As well, neutral polymorphisms in *gyrA*, which are not drug resistance conferring e.g. Glu21Gln, Thr80Ala, Ser95Thr, etc. are frequently observed in both FQ resistant and FQ susceptible isolates [44, 45]. The Thr80Ala polymorphism has been detected in African isolates [46] and it is regarded a lineage-specific marker for *M. tuberculosis* sub-lineage Uganda strains [44] that are prevalent in Uganda and in countries in central Africa [47]. In the current study, this polymorphism occurred in only “*M. tuberculosis* Uganda” isolates but further studies are required to demonstrate its usefulness as a marker for this genotype. Although neutral polymorphisms in resistance associated genes of *M. tuberculosis* are generally not resistance conferring [44], further research is required to ascertain they have no role in resistance. For example, the simultaneous occurrence of Thr80Ala and Ala90Gly polymorphisms in *gyrA/QRDR* led to a FQ hyper-susceptibility phenotype in laboratory-based experiments [32, 48, 49]. With the increase in sequencing data for *M. tuberculosis* isolates from across the globe, it would be interesting to investigate the frequency of isolates with dual mutations Thr80Ala & Ala90Gly in clinically circulating TB strains and ascertain the association between carriage of the dual mutations Thr80Ala & Ala90Gly with FQ hyper-susceptibility. These dual mutations occurred in two isolates in this study (TC84410 & TC84702) and they also occurred in nine clinical *M. tuberculosis* isolates in Congo-Brazzaville [50], implying they could be prevalent in Africa.

### Limitations

This study had certain limitations: First, we characterized MDR isolates that were resistant to second line anti-TB drugs from eSwatini, Somalia and Uganda however, the total number of isolates that met this criteria (*n* = 40) was small relative to the TB burden in those countries. As such, the true frequencies of drug resistance conferring mutations to second-line anti-TB drugs could be higher than we have reported if a larger sample size is used. However, the fact that the reported isolates were collected over a long period of time, studies with larger sample sizes require a lot of time. Second, our findings may not be generalizable especially for Somalia and Uganda as only a small fraction of M/XDR cases (10%-20%) are tested for resistance to second-line anti-TB drugs [23, 25]. Nevertheless, our findings provide key insight into the performance of molecular DST methods in detecting resistance to second-line anti-TB drugs in eSwatini, Somalia and Uganda.

## Conclusions

The frequencies of drug resistance conferring mutations to second-line anti-TB drugs in MDR isolates that are resistant to second line anti-TB drugs from eSwatini, Somalia and Uganda is high especially to FQs, implying that rapid molecular tests e.g. MTBDRplus/sl line probe assay (LPA) that has great promise in detecting MDR isolates [8], as well as DNA sequencing, are useful in detecting FQ and AMK/KAN/CAP resistant *M. tuberculosis* in those countries. Relatedly, the frequency of FQ resistance conferring mutations in *gyrB* was high relative to global estimates, and occurred independently of *gyrA* mutations implying that absence of *gyrB*/QRDR resistance mutations in panels for rapid molecular tests for detection of FQ resistance could lead to false negative results in this setting.

## Additional file

Additional file 1: **Table S1.** Sheet 1 –mutations in drug resistance associated genes/loci among MDR *M. tuberculosis* isolates from Uganda, Somalia and eSwatini that were resistant to any of: Ofloxacin, levofloxacin, moxifloxacin, kanamycin, capreomycin, amikacin; Sheet 2 –primers and sequences used. (XLSX 20 kb)

## Data Availability

The datasets used and/or analysed during the current study are available from the corresponding authors on reasonable request.

## Abbreviations

AMK: Amikacin
BSL-3: Biosafety Level 3
CAP: Capreomycin
DST: Drug susceptibility testing
FQs: Fluoroquinolones
KAN: Kanamycin
LPA: Line probe assay
LXF: Levofloxacin
MDR-TB: Multidrug resistant tuberculosis
MEGA6.06: Molecular Evolutionary Genetics Analysis Version 6.0
MIRU-VNTR: Mycobacterial Interspersed Repetitive Units-Variable Number of Tandem Repeats analysis
MXF: Moxifloxacin
NCBI: National Center for Biotechnological Information
OFX: Ofloxacin
QRDR: Quinolone-resistance determining region
*rrs*: 16S ribosomal RNA gene
RR-TB: Rifampicin resistant TB
TE: Tris-EDTA buffer solution, pH 8.0
XDR-TB: Extensively drug-resistant tuberculosis
